# Photocuring Hyaluronic Acid/Silk Fibroin Hydrogel Containing Curcumin Loaded CHITOSAN Nanoparticles for the Treatment of MG-63 Cells and ME3T3-E1 Cells

**DOI:** 10.3390/polym13142302

**Published:** 2021-07-14

**Authors:** Qingwen Yu, Zhiyuan Meng, Yichao Liu, Zehao Li, Xing Sun, Zheng Zhao

**Affiliations:** 1 State Key Laboratory of Advanced Technology for Materials Synthesis and Processing, Wuhan University of Technology, Wuhan 430070, China; 771101@whut.edu.cn (Q.Y.); mengzhiyuan@whut.edu.cn (Z.M.); lizehao@whut.edu.cn (Z.L.); sunxing@whut.edu.cn (X.S.); 2 Center for Evidence-Based and Translational Medicine, Zhongnan Hospital of Wuhan University, Wuhan 430070, China; yichaoliu619@whu.edu.cn

**Keywords:** curcumin, hyaluronic acid, silk fibroin, chitosan nanoparticle, MG-63 cells, MC3T3-E1 cells

## Abstract

After an osteosarcoma excision, recurrence and bone defects are significant challenges for clinicians. In this study, the curcumin (Cur) loaded chitosan (CS) nanoparticles (CCNP) encapsulated silk fibroin (SF)/hyaluronic acid esterified by methacrylate (HAMA) (CCNPs-SF/HAMA) hydrogel for the osteosarcoma therapy and bone regeneration was developed by photocuring and ethanol treatment. The micro or nanofibers networks were observed in the CCNPs-SF/HAMA hydrogel. The FTIR results demonstrated that alcohol vapor treatment caused an increase in β-sheets of SF, resulting in the high compression stress and Young’s modulus of CCNPs-SF/HAMA hydrogel. According to the water uptake analysis, SF caused a slight decrease in water uptake of CCNPs-SF/HAMA hydrogel while CCNPs could enhance the water uptake of it. The swelling kinetic results showed that both the CCNPs and the SF increased the swelling ratio of CCNPs-SF/HAMA hydrogel. The accumulative release profile of CCNPs-SF/HAMA hydrogel showed that the release of Cur from CCNPs-SF/HAMA hydrogel was accelerated when pH value was decreased from 7.4 to 5.5. Besides, compared with CCNPs, the CCNPs-SF/HAMA hydrogel had a more sustainable drug release, which was beneficial for the long-term treatment of osteosarcoma. In vitro assay results indicated that CCNPs-SF/HAMA hydrogel with equivalent Cur concentration of 150 μg/mL possessed both the effect of anti-cancer and promoting the proliferation of osteoblasts. These results suggest that CCNPs-SF/HAMA hydrogel with superior physical properties and the bifunctional osteosarcoma therapy and bone repair may be an excellent candidate for local cancer therapy and bone regeneration.

## 1. Introduction

Osteosarcoma is the most common primary malignant bone cancer in children and adolescents [1]. Nowadays, treatments of osteosarcoma comprise surgery and chemotherapy regimens. However, no less than 30% of patients still have resistance to chemotherapy treatment, finally surrendered to metastases, resulting in no substantial further improvement [2]. Moreover, these studies usually ignore the regeneration in bone tissue while treating osteosarcoma. Therefore, it is necessary to develop more effective drug delivery systems for osteosarcoma treatment.

Recently, natural hydrogel-based therapies are considered as attractive candidates to treat tumor due to their superior advantages including easy formulation, target injection, biodegradability, biocompatibility, as well as localized and sustained drug release profiles [3,4,5]. Moreover, hydrogels with water-rich 3D structures and similar components and structure of extracellular matrix (ECM) of target tissue provide space and mechanical stability for tissue regeneration [6,7]. Therefore, there is an urgent need to design hydrogels for treating osteosarcoma and promoting bone formation.

However, natural hydrogels commonly exhibit weak mechanical properties and fast degradation in vivo. To overcome the limitations of natural hydrogels, crosslinking strategy (i.e., physical or chemical) can be employed to stabilize the polymer network and, thereby, improve the physical properties of the hydrogel [8]. Photo-polymerization has gained significant interest for the synthesis of hydrogels due to its many advantages, including low reaction temperature, rapid solidification, simple and mild preparation process, low-cost production, and avoiding the use of organic solvent [9,10,11].

Amongst natural hydrogels, hyaluronic acid (HA), as one kind of natural polysaccharide, is the main component of the extracellular matrix and, thus, appealing for medical utility owing to its good biocompatibility and biodegradability. Furthermore, hyaluronic acid esterified by methacrylate (HAMA) hydrogel received attention for its low immunogenicity and biological activities [12,13]. Therefore, HAMA hydrogels have the potential for the osteosarcoma treatment and bone regeneration simultaneously. Unfortunately, photocuring HAMA gels generally have poor mechanical properties, fast degradation rate. This could make the hydrogel vulnerable to breakage when subjected to external forces, accelerating the drug release. Therefore, HAMA-based composite hydrogels need to be exploited.

Silk fibroin (SF) is a natural fibrous protein that is extracted from the cocoons of the Bombyx mori silkworm, has been widely used in hydrogel preparation for its unique mechanical properties, high biocompatibility, and controllable biodegradation rate [14,15]. Blending HAMA hydrogels with protein materials (such as silk fibroin) can mimic the composition and structure of the ECM and also improve strength and adjust degradation of HAMA hydrogel [16]. Therefore, developing novel HAMA/SF composite hydrogels could be necessary because it exhibits superior biocompatible, mechanical properties and bioactivity to promote tissue reconstruction.

In spite of their many favorable characteristics, hydrogel-based local tumor therapy also shows some disadvantages. The high water content of hydrogels often results in the relatively rapid drug release of hydrophilic drugs to the surroundings [17]. Moreover, the pore structure of hydrogel could lead to the initial burst release of low molecular weight drugs through diffusion [18,19,20]. Therefore, it is necessary to slow the diffusion rate of loaded drugs to extend the release profile of the drug delivery system (DDS) [21]. Recently, nanocarrier incorporated hydrogels were reported for efficient drug delivery [22]. The combination of drug-loaded nanoparticles and hydrogel-based delivery system can achieve high targeting via hydrogel and extend the duration of drug release by adopting nanoparticles.

Curcumin, a yellow-orange polyphenol compound derived from turmeric *Curcuma longa*, has received considerable interest in cancer therapeutics due to its anti-cancer activity. Moreover, it has demonstrated no toxicity to healthy organs at doses as high as 8 g/day in clinical trials [23,24,25]. Furthermore, a recent study revealed that Cur enhanced proliferation of osteoblasts and also induced osteogenesis-related gene expressions [26,27,28,29]. Unfortunately, the poor solubility and bioavailability limit its use in clinical treatment.

A variety of nanoparticle carriers have been developed to enhance solubility and bioavailability of curcumin [30]. Chitosan, a biopolymer consisting of β-(1,4)-linked D-glucosamine and N-acetyl-D-glucosamine, was preferred in the preparation of nanoparticles for its degradability and biocompatibility. Besides, Cur loaded CS nanoparticles (CCNP) have been developed to treat different cancers such as breast, colon, and lung owing to good biodegradability, biocompatibility, and non-toxicity [31]. However, the studies using CCNPs to expand the duration of drug release in osteosarcoma treatment and the bone regeneration is seldom.

Herein, we report a nanocarrier incorporated hydrogel consisting of HAMA/SF composite hydrogels and CCNPs for localized osteosarcoma chemotherapy and bone regeneration. We prepared HAMA/SF composite hydrogels by photocuring followed by alcohol vapor treatment. The morphology, size, surface zeta potential of nanoparticle and Cur loading efficiency, in vitro on-demand release of hydrogel/nanoparticle were characterized. Besides, the water uptake, swelling rate and compression strength of hydrogel were also characterized in this study. The therapy efficacy of CCNPs-SF/HAMA hydrogel in anti-osteosarcoma and osteoblast proliferation was further investigated by in vitro experiments. This work provides a novel combination therapy in hydrogel platform to promote the apoptosis of osteosarcoma cells and the proliferation of osteoblasts.

## 2. Materials and Methods

### 2.1. Materials

Hyaluronic acid (97%, 40–100 Kda) was obtained from Macklin Co (Wuhan, China). Methacrylic anhydride (94%, containing 0.2% topanol stabilizer) was purchased from Macklin Co (Wuhan, China). 2-Hydroxy-4′-(2-hydroxyethoxy)-2-methylpropio-phen one (I2959) was purchased from Aladdin Co (Shanghai, China). Sodium tripolyphosphate (TPP) (AR, 98%) was purchased from Aladdin Co (Wuhan, China). Chitosan (Deacetylation degree ≥95%, viscosity of 100–200 mpa. s) was obtained from Aladdin Co (Wuhan, China). Curcumin (368.38 (MW)) was obtained from Aladdin Co (Wuhan, China). MTS dye was brought from Promega Biotech Co. Ltd. (Beijing, China). Calcein/PI Cell Viability/Cytotoxicity Assay Kit was purchased from Beyotime Biotech Inc.

### 2.2. Preparation of Pure SF and HAMA

The pure silk fibroin was prepared following previous study [32]. Briefly, to remove the sericin, silkworm cocoons were boiled in Na_2_CO_3_ solution for three hours. Next, the degummed silk fibers were dissolved in a ternary solution at 80 °C (CaCl_2_:CH_3_CH_2_OH:H_2_O = 1:8:2, molar ratio). Pure SF solution was prepared by dialyzing, and the pure silk fibroin was obtained by lyophilization.

The HAMA were fabricated by side group modification. Briefly, HA was dissolved in deionized water at the concentration of 0.01 g/mL and then it was cooled at 4 °C. Subsequently, methacrylic anhydride and NaOH (5 M) were added to the solution, and was slowly stirred for a day. HAMA was obtained by dialyzing and lyophilization.

### 2.3. Preparation of CCNPs

The chitosan nanoparticles (CSNP) were prepared based on electrostatic interaction with poly-anionic sodium tripolyphosphate (TPP). Briefly, chitosan was dissolved in acetic acid solution (1%) at the concentration of 1 mg/mL and the pH was adjusted to 4.5 through NaOH solution (5M). TPP was dissolved in deionized water (1 mg/mL) and then TPP solution was dropped into the chitosan solution with stirring (CS:TPP = 5:1 (m/m)). Finally, the CSNPs were obtained by centrifugation at 9000 rpm for 30 min. For CCNPs preparation, after adding the TPP solution, subsequently, Cur (2 mg/mL in ethanol) was dispersed into the complex solution and then the CCNPs were obtained by sonicating in an ice bath followed by centrifugation (9000 rpm, 30 min).

### 2.4. Preparation of CCNPs-SF/HAMA Hydrogels and SF/HAMA Hydrogels

**Scheme 1:**
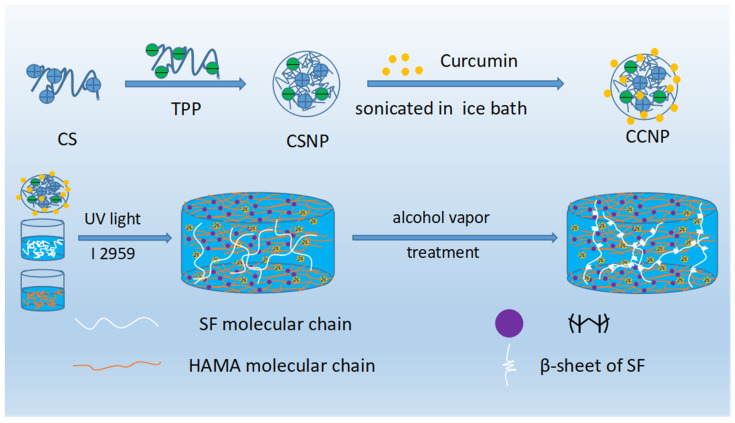
Fabrication of CCNPs and CCNPs-SF/HAMA hydrogel.

For preparation of SF/HAMA hydrogel, Irgacure 2959 (I2959) was dissolved in PBS at a concentration of 0.1% (*w*/*v*). HAMA was dissolved in PBS/I2959 solution at a concentration of 5% (*w*/*v*). SF was also dissolved in PBS/I2959 solution at a concentration of 5%/10% (*w*/*v*). Then, the HAMA solution (5% (*w*/*v*)) and SF solution (5%/10% (*w*/*v*)) was mixed in the proportion of 1:1 (*w*/*v*). Finally, the Photocrosslinked SF/HAMA hydrogel was prepared by exposing the mixture to ultraviolet (UV) light (365 nm, UPF100, Uvata) for 3 min. For preparation of CCNPs-SF/HAMA hydrogel, CCNPs was dispersed into PBS/I2959 solution prepared before, then the SF was dissolved in the PBS/I2959/CCNPs solution at the concentration of 5% or 10% (*w*/*v*). CCNPs-SF/HAMA hydrogel was prepared as the method we described before. Detail of fabrication process was shown in Scheme 1.

### 2.5. Characterization of CCNPs

CCNPs were obtained in different mass ratio of curcumin and chitosan. For the determination of the loading content, CCNPs were dissolved in acetic acid and then were further dissolved in ethanol. The Cur in the solution was measured according to the optical density at 425 nm. Optical density of CSNPs solution was also measured for control. The drug loading ratio was calculated as the following equation:

(1) $$\mathrm{Drug}\;\mathrm{loading}\;\mathrm{ratio}=\frac{\mathrm{Cur}\;\mathrm{in}\;\mathrm{the}\;\mathrm{solution}}{\mathrm{nanoparticles}\;\mathrm{weight}}\times 100\%$$

The particle size distribution and the zeta potential of CCNPs were measured by Malvin laser particle size analyzer. The morphology was observed with transmission electron microscopy (TEM H-7000FA, Hitachi, Japan).

### 2.6. Morphology and Pore Structure of CCNPs-SF/HAMA Hydrogels and SF/HAMA Hydrogels

SF, HAMA, and SF/HAMA Hydrogels were frozen at −20 °C and then lyophilized. The freeze-dried hydrogels were observed with scanning electronic microscopy (SEM, S-4800, Hitach, Japan). The SEM images were analyzed using the software “Image J” to evaluate the pores of the sample.

### 2.7. The Swelling Behavior and the Water Uptake of CCNPs-SF/HAMA Hydrogels and SF/HAMA Hydrogels

Briefly, the wet weight of CCNPs-SF/HAMA hydrogels and SF/HAMA hydrogels were measured (W_w_) and then incubated in PBS solution at 37 °C. At the dedicated time intervals, the weight of swelled hydrogel was measured (Ws). The swelling ratio (SR) was determined by the following formula:

(2) $$\mathrm{SR}\;(\%)\;=\frac{W_{s}-W_{w}}{W_{w}}\times 100$$

The water uptake of the CCNPs-SF/HAMA hydrogels and SF/HAMA hydrogels were also measured. Briefly, freeze-dried CCNPs-SF/HAMA hydrogels (W_d_) were incubated in distilled water at 37 °C for 4 h. Then, the weight of the hydrogels (W_s_) was measured. The water uptake (Q) of the CCNPs-SF/HAMA hydrogels and SF/HAMA hydrogels were calculated as the following equation:

(3) $$Q\;(\%)\;=\frac{W_{s}-W_{d}}{W_{d}}\times 100$$

### 2.8. Compression Strength of CCNPs-SF/HAMA Hydrogels

To further explore the compression strength of SF/HAMA hydrogels with different SF content, we fabricated cylindrical hydrogels. The compressive strength was characterized by a Universal Testing System (Instron 5967, China). Young’s modulus at the fracture was also calculated.

### 2.9. Cur Release of the CCNPs and CCNPs-SF/HAMA Hydrogels

A total of 5mg CCNPs or CCNPs-SF/HAMA hydrogel (containing 5 mg CCNPs) was immersed in different PBS solutions (PBS, pH 5.5 tween 80 (1%)or PBS, pH 7.4, tween 80 (1%)). They were poured into a dialysis bag and then immersed in 50 mL of different PBS. At the dedicated time intervals, 5 mL of the supernatant was replaced with 5 mL fresh PBS. The loading rate of Cur in the PBS was measured according to the optical density at 425 nm.

### 2.10. Cell Culture

Human osteosarcoma cells (MG-63) and mouse pre-osteoblast cells (MC3T3-E1) were obtained for the in vitro biological assessment. The MG-63 and MC3T3-E1 cells were obtained from China Center for Type Culture Collection (Wuhan, China). MG-63 cells were cultured with MEM containing 10% fetal bovine serum (FBS) and 1% penicillin/streptomycin (P.S). MC3T3-E1 cells were cultured in α-MEM with 10% FBS and 1% P.S. The cells were then incubated in a humidified atmosphere (95% air and 5% CO_2_) at 37 °C.

### 2.11. Cytotoxicity and In Vitro Chemotherapy EFFECT

Both anti-cancer effect of the CCNPs-SF/HAMA hydrogel on MG-63 cells and proliferation effect of MC3T3-E1 cells were evaluated through MTS assay. Briefly, MG-63 and MC3T3-E1 cells were co-cultivated with CCNPs-SF/HAMA hydrogel in the 48-well plates (5 × 10^3^ cells/well) for 48 h, respectively. Then, 1 mL of the MTS/medium solution (1:1 (*w*/*v*)) was added. After incubating for 4 h, the optical density was measured at 490 nm. Cell viability was calculated by the following formula:

(4) $$\mathrm{Cell}\;\mathrm{viability}\;(\%)\;=\frac{\mathrm{Absorbence}\;\mathrm{of}\;\mathrm{test}\;\mathrm{cells}}{\mathrm{Absorbence}\;\mathrm{of}\;\mathrm{control}}\times 100$$

Moreover, after treated with CCNPs-SF/HAMA hydrogel for 48 h, cells were stained with the Calcein-AM/PI Double Stain Kit to observed the live cells. The morphology and growth states were observed and recorded on an inverted fluorescent microscope.

### 2.12. Statistical Analysis

The statistical analysis was performed using one-way analysis of variance (ANOVA) followed by Tukey’s multiple comparison test to determine any significant differences among different groups tested. Additionally, a *t*-test (non-parametric) was used when evaluating the statistical significance between only two groups. The significant levels were marked with (*) for *p* < 0.05, (**) for *p* < 0.01, and (***) for *p* < 0.001.

## 3. Results

### 3.1. Characterization of CCNPs

**Figure 1:**
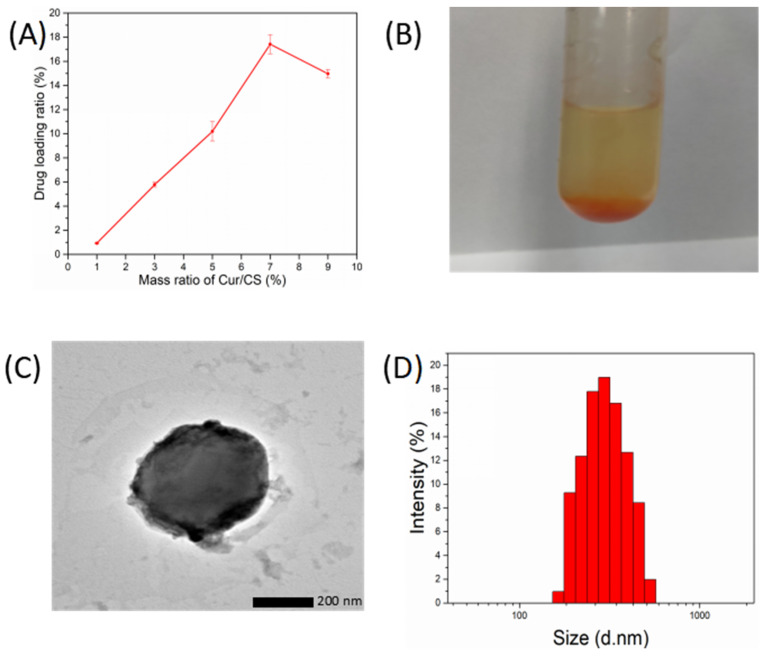
Drug loading ratio of CCNPs (**A**), CCNPs aggregated in distilled water (**B**), TEM images of CCNPs (**C**). size distribution of CCNPs (**D**).

As shown in the Figure 1A, with the increase in curcumin, the drug loading ratio of CCNPs first increased and then decreased. The drug loading ratio reached a maximum of 18% when the mass ratio of Cur and CS was 7%. However, CCNPs are inclined to aggregate together when the mass ratio of Cur and CS was above 5% (Figure 1B). Therefore, the dispersed CCNPs with a drug loading ratio of 10% when the mass ratio of Cur and CS was 5% were obtained for further analysis and preparation of CCNPs-SF/HAMA hydrogels.

As shown in the TEM image (Figure 1C) and size distribution of the CCNPs (Figure 1D), the CCNPs showed spherical shape with an average diameter of about 400 nm and a polydispersity index (PDI) of 0.24. As shown in Table S1, the zeta potential of CCNPs was 16.57 ± 1.13 mV. The nanostructure and positive charge of CCNPs can accelerate cellular uptake and are beneficial for tumor treatment [33]. Obviously, the loading of curcumin led to a decrease in zeta potential of CSNPs, which might reduce their stability, and finally led to agglomeration.

### 3.2. FTIR Spectra of CCNPs and CCNPs-SF/HAMA Hydrogels

**Figure 2:**
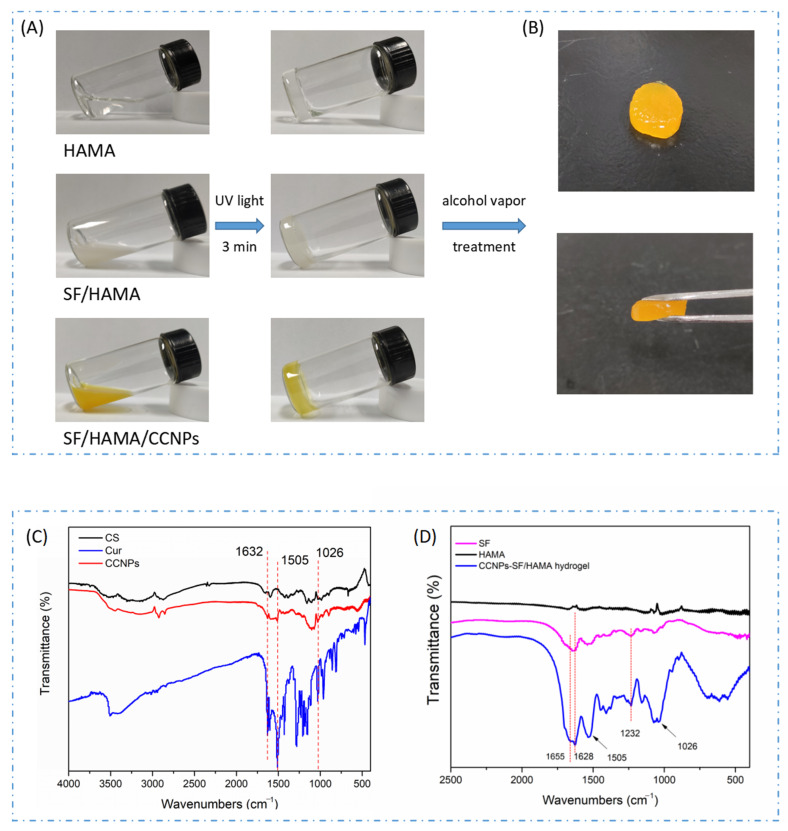
The digital picture of HAMA, HAMA/SF, CCNPs-SF/HAMA hydrogels before and after UV treatment (**A**), The digital picture of CCNPs-SF/HAMA hydrogel after alcohol vapor treatment (**B**), the FTIR spectra of CCNPs (**C**), and CCNPs-SF/HAMA hydrogel (**D**).

The HAMA, HAMA/SF, and CCNPs-SF/HAMA hydrogels were obtained by photocuring and alcohol vapor treatment (Figure 2A,B). Fourier infrared spectroscopy was used to verify the loading of Cur and analyze the conformational transformation of SF (Figure 2C,D).

The characteristic peaks around 1050–1000 cm^−1^, 1690–1630 cm^−1^, and 1500 cm^−1^ were attributed to =C-O-C, the stretching vibration of C=C, and skeleton vibration of substituted benzene ring of Cur, respectively. As shown in the Figure 2A, the characteristic peaks of the CCNPs around 1505 cm^−1^, 1026 cm^−1^, and 1632 cm^−1^, indicating that Cur was successfully loaded on the CS nanoparticles. Besides, characteristic peaks of Cur (1505 cm^−1^ and 1026 cm^−1^) were found in CCNPs-SF/HAMA hydrogel. These results demonstrated that CCNPs were loaded into the SF/HAMA hydrogel successfully.

The characteristic peaks around 1620–1635 cm^−1^ and 1230–1235 cm^−1^ represented β-sheet structure of SF. Characteristic peaks around 1650–1655 cm^−1^ were attributed to the α-form of SF [34,35,36,37]. As shown in Figure 2B, the characteristic peaks of the CCNPs-SF/HAMA hydrogel appeared around 1655 cm^−1^, were attributed to α-helix of SF, and the band of 1628 cm^−1^ and 1232 cm^−1^, represented β-sheet structures of SF. Besides, comparing with SF, the band of CCNPs-SF/HAMA hydrogel around 1655 cm^−1^ weaken, and the band of CCNPs-SF/HAMA hydrogel around 1628 cm^−1^ and 1232 cm^−1^ became stronger. These results demonstrated that alcohol vapor treatment resulted in the increase in β-sheets of SF.

### 3.3. Morphology and Pore Structure of CCNPs-SF/HAMA Hydrogels

**Figure 3:**
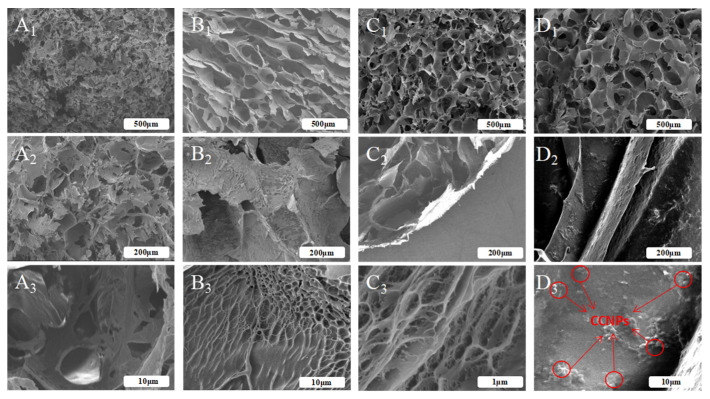
The cross section SEM images of the freeze-dried SF scaffold (**A_1_**,**A_2_**,**A_3_**), SF/HAMA (**B_1_**,**B_2_**,**B_3_**), HAMA (**C_1_**,**C_2_**,**C_3_**), and CCNPs-SF/HAMA freeze-dried hydrogels (**D_1_**,**D_2_**,**D_3_**).

In the present work, lyophilized SF scaffold, and HAMA, SF/HAMA, CCNPs-SF/HAMA lyophilized hydrogel were fabricated for morphological studies [38]. Their SEM images were shown in Figure 3. In general, SEM images of all the samples demonstrated typically porous structure (Figure 3A_1_,B_1_,C_1_,D_1_). However, the micro or nanofibers networks were established in the hydrogels with HAMA (Figure 3B_3_,C_3_), while pure SF hydrogel was bare (Figure 3A_3_). The results suggested that the HAMA contributed to the formation of the micro or nanofibers networks of HAMA, SF/HAMA hydrogels, as well as CCNPs-SF/HAMA hydrogel. Moreover, CCNPs were clearly observed on the wall of CCNPs-SF/HAMA lyophilized hydrogel and they were dispersed homogeneously inside the SF/HAMA hydrogel (Figure 3D_3_).

**Figure 4:**
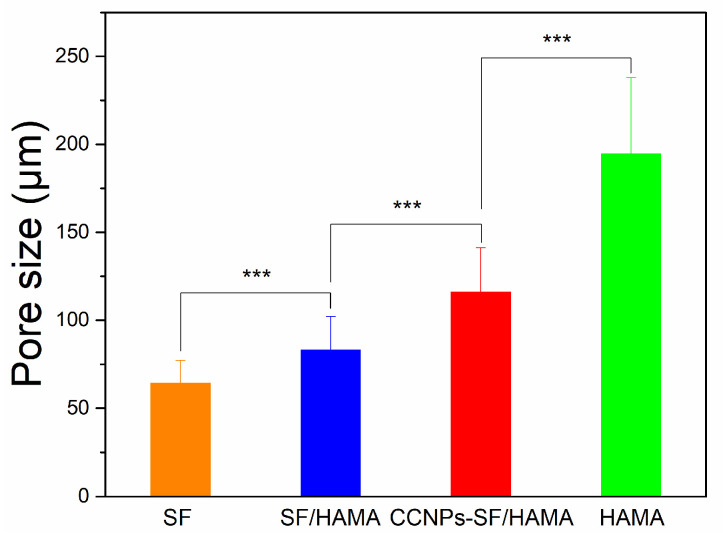
Pore size of CCNPs-SF/HAMA hydrogels *** *p* < 0.001 are differences between the indicated groups.

Interestingly, pore size determination carried out on the SEM images of hydrogels using Image J analysis (Figure 3) indicated a decrease in mean pore diameter with the addition of SF content. As shown in Figure 4, the pore size of the pure HAMA hydrogel was about 195 μm while the SF/HAMA hydrogel and pure SF freeze-dried scaffold were 83 μm, 64 μm, respectively. The value of pore size was associated with the water binding capacity of the hydrogels during freeze-drying process [39,40,41,42]. After freeze drying, the higher porosity and bigger pore size would be generated in the hydrogel with more water [43]. It indicated that SF might cause a decrease in water retention ability of CCNPs-SF/HAMA hydrogel. Besides, the pore size of CCNPs-SF/HAMA hydrogel was higher than SF/HAMA hydrogel, indicating that CCNPs could enhance the water uptake of CCNPs-SF/HAMA hydrogels.

### 3.4. Analysis of Water Uptake, Swelling Ratio of CCNPs-SF/HAMA Hydrogels

**Figure 5:**
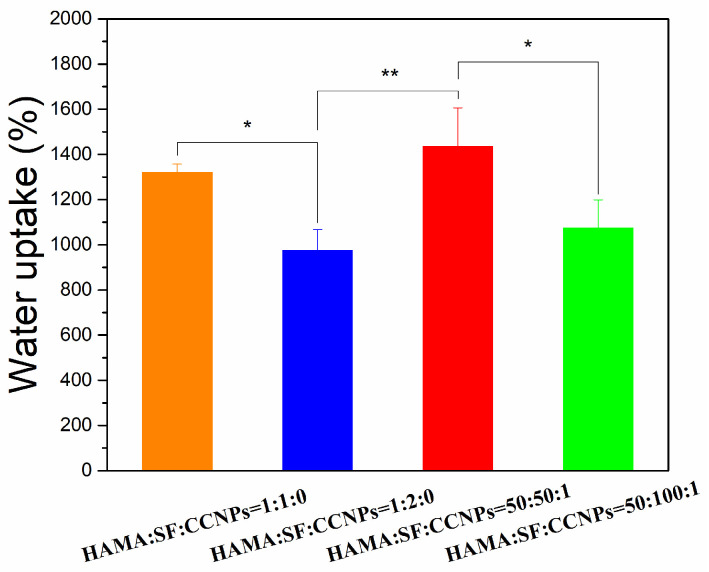
Water uptake of CCNPs-SF/HAMA hydrogels. Data are expressed as mean ± SD (*n* = 4); * *p* < 0.05, and ** *p* < 0.01 are differences between the indicated groups.

To further explore the effect of SF on the water retention of CCNPs-SF/HAMA hydrogels, we fabricated CCNPs-SF/HAMA hydrogels and SF/HAMA hydrogels in different mass ratios of HAMA and SF (HAMA/SF = 1:1,1:2 (*w*/*w*)). Their water uptake was shown in Figure 5. Both the CCNPs-SF/HAMA hydrogels and SF/HAMA hydrogels showed a higher water uptake when the mass ratio of HAMA and SF was 1:1. Besides, the water uptake of CCNPs-SF/HAMA hydrogels was higher than SF/HAMA hydrogels when the mass ratio of HAMA and SF was either 1:1 or 1:2. The results indicated that SF caused a decrease in water uptake of CCNPs-SF/HAMA and SF/HAMA hydrogels while CCNPs could enhance the water uptake of hydrogels. These results are in accord with the reported morphology studies (Figure 3).

**Figure 6:**
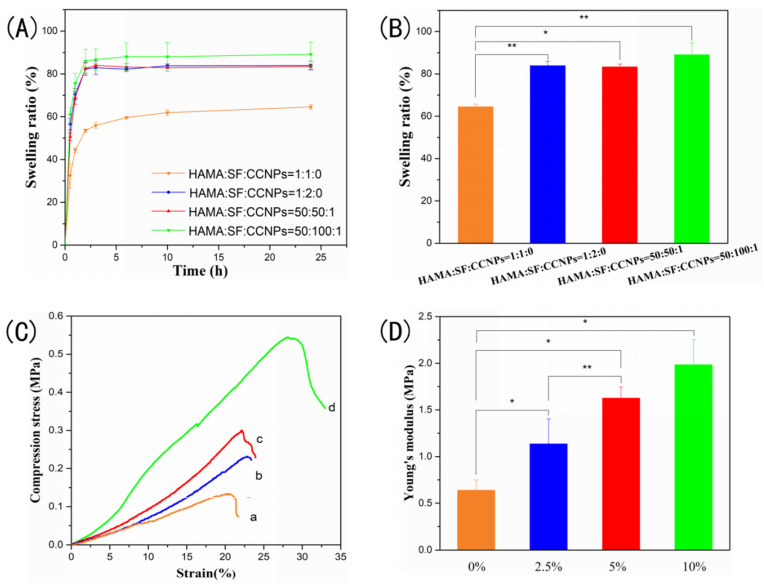
Swelling ratio curve (**A**) and swelling ratio (**B**) of CCNPs-SF/HAMA hydrogels, stress-strain curve and Young’s modulus of CCNPs-SF/HAMA hydrogels with different concentrations (0% (a), 2.5% (b), 5% (c), 10% (d)) of SF (**C**,**D**). Data are expressed as mean ± SD (*n* = 4); * *p* < 0.05, and ** *p* < 0.01 are differences between the indicated groups.

The swelling ability of CCNPs-SF/HAMA hydrogels and SF/HAMA hydrogels with different mass ratios of HAMA and SF were also monitored in SBF buffer owing to evaluation of their application in tissue engineering (Figure 6A,B). As shown in Figure 6A, an increase in swelling ratio of CCNPs-SF/HAMA hydrogel was observed in comparison to SF/HAMA hydrogel. It might be attributed to the hydrophilic groups of CCNPs. Moreover, both the CCNPs-SF/HAMA and SF/HAMA hydrogel showed a higher swelling ratio when the mass ratio of HAMA and SF was 1:2. Owing to the increase in β-sheet structures, CCNPs-SF/HAMA and SF/HAMA hydrogels tended to shrink and lose water in the presence of alcohol vapor. They absorbed water again to restore the initial shape when immersed in SBF buffer, which resulted in a higher swelling ratio of hydrogels. Therefore, the hydrogel with higher SF content represented a higher swelling ratio owing to more β-sheets structures of SF. In summary, both the CCNPs and the SF content increased the swelling ratio of CCNPs-SF/HAMA hydrogel, which is beneficial for the adherence between hydrogel and tissue.

### 3.5. The Compression Stress of CCNPs-SF/HAMA Hydrogels

In order to further investigate the effect of SF content on the mechanical stability of hydrogels, compression strength of CCNPs-SF/HAMA hydrogels with different concentrations (0%, 2.5%, 5%, 10% (*w*/*w*)) of SF were characterized. The fracture strength of hydrogels showed a non-linear relationship with SF content (Figure 6C), indicating that their ability to resist deformation was enhanced with SF content increased. Figure 6D shows Young’s modulus of the CCNPs-SF/HAMA hydrogels. The CCNPs-SF/HAMA hydrogel showed higher Young’s modulus than HAMA hydrogel, suggesting that rigidity was also reinforced with the addition of SF. It might be attributed to the increase in β-sheet structure of SF.

In conclusion, the CCNPs-SF/HAMA hydrogels with SF concentration of 10% (mass ratio of HAMA and SF was 1:2) were chose to do further research for their excellent water uptake, suitable swelling ratio, and superior mechanical property.

### 3.6. pH-Responsive Release of CCNPs-SF/HAMA Hydrogel

**Figure 7:**
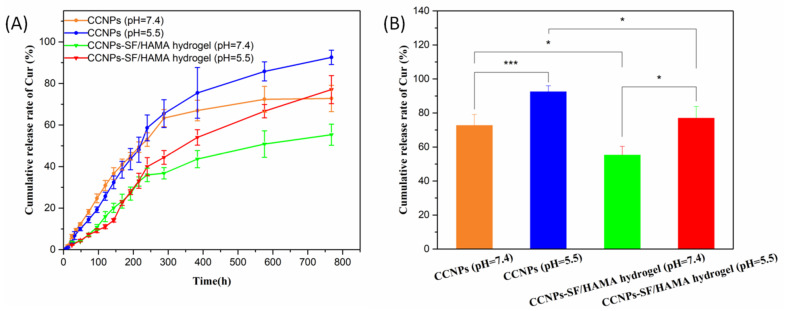
Cur release of the CCNPs and CCNPs-SF/HAMA hydrogels in different pH (**A**). Cumulative release of the CCNPs and CCNPs-SF/HAMA hydrogels in 32 days (**B**). Data are expressed as mean ± SD (*n* = 4); * *p* < 0.05, and *** *p* < 0.001 are differences between the indicated groups.

The release kinetics of Cur from CCNPs-SF/HAMA hydrogel in PBS buffer at different pH values were shown in Figure 7A. The data showed that the release of Cur from CCNPs-SF/HAMA hydrogel was slow and sustained and was accelerated when pH value was decreased from 7.4 to 5.5. The cumulative release rate of Cur from the CCNPs reached 92.6% at pH 5.5 and 72.8% at pH 7.4 in 32 days, respectively. On the other hand, CCNPs was compared with CCNPs-SF/HAMA hydrogel to investigate drug release profile (Figure 7A). The cumulative release rate of Cur from the CCNPs-SF/HAMA hydrogel was 77.1% at pH 5.5 and 55.3% at pH 7.4 in 32 days (Figure 7B). The comparison of drug release results for CCNPs and CCNPs-SF/HAMA hydrogel illustrated that CCNPs-SF/HAMA hydrogel had lower drug release rate and it was more sustainable.

At pH < 6, the ionizable amino groups on the surface of CCNPs are protonated, making chitosan a cationic polyelectrolyte. Subsequently, the CS start to swell due to the repulsion of the polymeric chains. Consequently, the release of Cur was accelerated. The pH-responsive drug release behavior made CCNPs-SF/HAMA hydrogel safer in the bloodstream (pH 7.4) with more effective release at the tumor sites (pH 4–6). Moreover, the combination of hydrogel could delay the Cur release of CCNPs. It is beneficial for the long-term treatment of osteosarcoma. Thus, the extended and pH-triggered drug release behavior made CCNPs-SF/HAMA hydrogels an excellent candidate for in vivo cancer therapy.

### 3.7. In Vitro Anti-Cancer and Osteoblast Proliferation Efficiency of CCNPs-SF/HAMA Hydrogel

**Figure 8:**
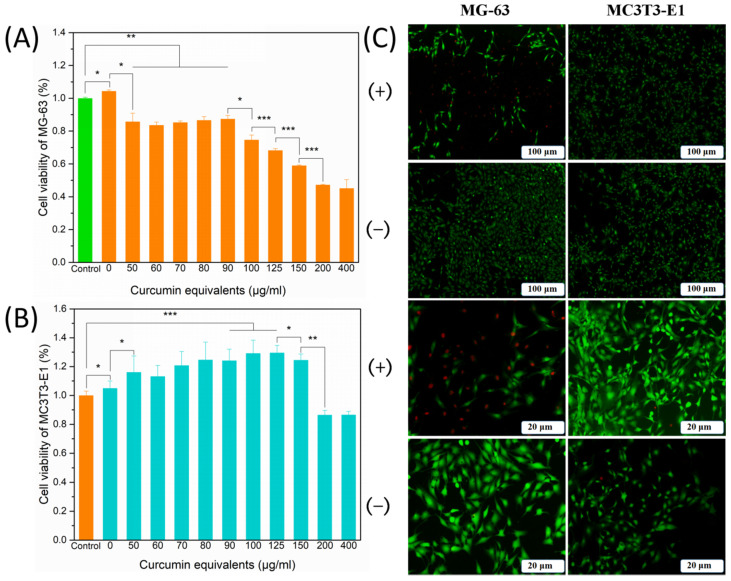
Cell viability of MG-63 (**A**) and MC3T3-E1 (**B**) cells after incubation with CCNPs-SF/HAMA hydrogels with different loading content for 48 h. Live and dead staining assay of cells with or without CCNPs-SF/HAMA hydrogels (**C**). Data are expressed as mean ± SD (*n* = 6); * *p* < 0.05, ** *p* < 0.01, and *** *p* < 0.001 are differences between the indicated groups.

In vitro anti-cancer effects of CCNPs-SF/HAMA hydrogels with equivalent Cur concentration ranging from 0 to 400 μg/mL on MG-63 cells were shown in Figure 8A. CCNPs-SF/HAMA hydrogels exhibited no obvious toxicity to MG-63 cells with the increase in the equivalent Cur concentration from 0 to 90 μg/mL. When the concentration was increased from 90 to 400 μg/mL, the cell survival rate decreased from 87.4% to 45.1%. These results indicated that CCNPs-SF/HAMA hydrogels are highly dose-sensitive hydrogels.

Furthermore, in vitro proliferative response of CCNPs-SF/HAMA hydrogels with different equivalent concentration of Cur on osteoblasts were shown in Figure 8B. When the equivalent concentration of Cur was less than 150 μg/mL, the viability of MC3T3-E1 cells could be improved by CCNPs-SF/HAMA hydrogels, varying from 104.9% to 124.5% with the increase in the equivalent Cur concentration from 0 to 150 μg/mL, respectively. When the concentration was increased from 150 to 400 μg/mL, the cell survival rate decreased from 124.5% to 86.6%. These results showed that CCNPs-SF/HAMA hydrogels with an equivalent Cur concentration of 150 μg/mL had obvious anti-cancer activity and could promote the osteoblasts proliferation. Therefore, the CCNPs-SF/HAMA hydrogels with an equivalent Cur concentration of 150 μg/mL were chosen to investigate the growth state of cells.

Live and dead cells stained with fluorescent dye at different magnifications were observed under a microscope to further study the growth state of cells cultured with the CCNPs-SF/HAMA hydrogel (Figure 8C). The MC3T3-E1 cells exhibited a morphology and growth state in the CCNPs-SF/HAMA hydrogels group similar to that in the control group. However, the MG-63 cells were effectively killed in the presence of CCNPs-SF/HAMA hydrogels, while there were almost no dead cells in the control group. These results demonstrated the excellent in vitro anti-osteosarcoma therapy and bone proliferation effect of the CCNPs-SF/HAMA hydrogels.

## 4. Conclusions

In summary, we constructed a nanocarrier incorporated hydrogel for favorable physical properties, sustainable, and pH-controlled drug release, anti-osteosarcoma ability, and tumor-causing bone defect repair. The β-sheet structures of SF were formed in CCNPs-SF/HAMA hydrogel through alcohol vapor treatment. The increased β-sheet structures of SF reinforced mechanical property but decreased water retention ability of CCNPs-SF/HAMA hydrogel. Owing to the hydrophilicity of HAMA and CCNPs, CCNPs-SF/HAMA hydrogel still possessed an excellent water up take of 1075%. Meanwhile, the CCNPs-SF/HAMA hydrogel exhibited pH-responsive release of Cur making CCNPs-SF/HAMA hydrogel safer in the bloodstream (pH 7.4) with more effective release at the tumor sites (pH 4–6). The CCNPs-SF/HAMA hydrogel showed a dose-dependent inhibition effect on the growth of MG-63 cells. Moreover, the CCNPs-SF/HAMA hydrogel could obviously promote the proliferation of MC3T3-E1 cells in vitro. Therefore, the CCNPs-SF/HAMA hydrogel with superior physical properties and the dual function of osteosarcoma therapy and bone regeneration may be an excellent candidate for local cancer therapy and bone regeneration.

## Data Availability

Not applicable.

## Supplementary Materials

The following are available online at https://www.mdpi.com/article/10.3390/polym13142302/s1, Table S1: The particle size, PDI, and zeta potential of CSNPs and CCNPs.
