# Supplementary material for: Photocuring Hyaluronic Acid/Silk Fibroin Hydrogel Containing Curcumin Loaded CHITOSAN Nanoparticles for the Treatment of MG-63 Cells and ME3T3-E1 Cells
Source: Polymers (Basel). 2021 Jul 14;13(14):2302. doi: 10.3390/polym13142302 (PMC8309346; doi:10.3390/polym13142302)
Supplement: Supplementary file 1 [file polymers-13-02302-s001.zip › polymers-1219928-supplementary.pdf]

Table. S1 The particle size distribution, PDI and zeta potential of CSNPs and CCNPs

Particles	Particle Size (nm) ±SD	Polydispersity (PDI)	Zeta-potential (mV) ± SD
CSNPs	374.67 ± 10.57	0.26 ± 0.05	56.37 ± 1.06
CCNPs	399.67 ± 12.23	0.24 ± 0.03	16.57 ± 1.13
